# CODE beyond FAIR: a roadmap for reusable research software

**DOI:** 10.1038/s41597-026-06705-6

**Published:** 2026-03-12

**Authors:** Roberto Di Cosmo, Sabrina Granger, Konrad Hinsen, Nicolas Jullien, Daniel Le Berre, Violaine Louvet, Camille Maumet, Clémentine Maurice, Raphaël Monat, Nicolas P. Rougier

**Affiliations:** 1 Inria Paris, Paris, France; 2 Université Paris Cité, Paris, France; 3 Inria Lyon, Lyon, France; 4 Centre de Biophysique Moléculaire (CNRS), Orléans, France; 5 Synchrotron SOLEIL, Saint Aubin, France; 6 IMT Atlantique, Brest, France; 7 Univ. Artois, CNRS, UMR 8188, CRIL, Lens, France; 8 Univ. Grenoble Alpes, CNRS, Grenoble INP (Institute of Engineering Univ. Grenoble Alpes), LJK, 38000 Grenoble, France; 9 Inria, Univ Rennes, CNRS, Inserm, Rennes, France; 10 Univ. Lille, CNRS, Inria, UMR 9189 CRIStAL, Lille, France; 11 Centre Inria de l’université de Bordeaux, Bordeaux, France

**Keywords:** Research data, Research management, Policy

## Abstract

FAIR principles are a set of guidelines aiming at simplifying the distribution of scientific *data* to enhance reuse and reproducibility. This article focuses on *research software*, which significantly differs from data in its living nature, and its relationship with free and open-source software. We provide a tiered roadmap to improve the state of research software, which takes into account the full range of stakeholders in the research software ecosystem: all scientific staff – regardless of prior software engineering training – but also institutions, funders, libraries and publishers.

## Background & Summary

### Open research software as a pillar of research

Research software has now become essential in all areas of scientific research, both as a research tool, as a research product, and as a research object. In the biology literature, Howison *et al*.^1^ established in 2014 that more than a third of research articles cited software. In the largest case study we are aware of, Bassinet *et al*.^2^ automatically processed over 908,000 research articles, and found that the percentage of articles mentioning the use of software rose from 33% in 2013 to 48% in 2021. A follow-up analysis showed that only 10% of research software developed in France uses proprietary licenses^3^. Research software has emerged as a scientific output of equal importance to publications and data in the open science perspective^4^.

### Research software diversity

Research software plays both a central and critical role in almost every aspect of modern science. Its usage varies heavily^5^: it can be used to process data, perform analyses, model complex phenomena, drive various apparatus, design surveys, setup experiments, write documents, and it can itself be a topic of research. This list has dramatically expanded during the last few decades, to the point that it would be now extremely difficult or impossible to conduct research without proper software. These pieces of software can take many different forms, ranging from a short script to a binary executable created from millions of lines of source code. If we were to scrutinize more closely all this software, we would discover an ever greater variety. According to the historical encyclopaedia of programming languages, there exist approximately 9,000 programming languages^6^. Most of them are hardly used outside specific niches, whereas a few languages are dominant. To name just a few, we mention C/C++, Fortran, Matlab, Python, Julia and R that seem to dominate the contemporary scientific landscape. If we now consider the cross product of language, form, usage and epistemic diversity, we get a small glimpse of the many forms of scientific software and their immense diversity.

### Research software: a living nature

This diversity is comparable to some extent to research diversity. However, unlike many other scientific objects (research papers & data), software packages can be complex living entities that are continuously evolving under the direct action of core developers and various contributors^7^. This means that there does not exist something like a single final state that could be considered the *version of record* to be archived once and for all for later reuse. It is actually not rare to have many versions of the same software (e.g. stable, unstable, latest, deprecated, etc.) that co-exist for some specific reasons. Software evolves and continues to do so as long as there is one person willing to contribute some code, tests, documentation, bug reports or even simple ideas. This mutable nature makes it a quite singular object in the research landscape, requiring a *record of versions* rather than the *version of record* which is central for journal articles. Furthermore, software cannot be easily separated from its biotope, which corresponds to the operating systems it runs on, taking advantage of the huge stack of software libraries that possess their very own lives. No environment is like another, such that the smallest change can have dramatic effects on the re-usability, or worse, on the correctness of a piece of software^8^. This makes preserving software while maintaining its functionalities quite a tricky operation.

### A porous frontier

Software development is less than a century old, but it has already undergone a series of dramatic changes. One of them is the explosive growth of *open source*, which has taken by storm the software industry that was traditionally based on *closed source*. Open source means that the source code of a piece of software is made freely available for possible modification and redistribution. This is not the case of closed source software, which is generally distributed as non-modifiable binary executables. The actual term “open source” has been popularized by Eric S. Raymond and Bruce Perrens at the end of the 1990s^9^, even though the free software movement founded by Richard Stallman is more than a decade older^10^. The Free/Libre Open-Source Software (FLOSS) community had to develop from the start good practices on how to find, access, evolve and re-use software, and has faced for decades issues like governance, recognition and sustainability^11^. FLOSS has a global scope, with academia having been only a small part of it since the beginning.

### Open Science to the rescue, but

In 2016, Wilkinson *et al*.^12^ published the article “The FAIR Guiding Principles for scientific data management and stewardship that encouraged academia, industry, funding agencies, and scholarly publishers to endorse a set of principles to promote the reuse of scholarly data. These FAIR principles (for Findability, Accessibility, Interoperability, and Reusability) have since then become highly popular in academia. A majority of stakeholders have adopted these principles at various levels. The FAIR principles were clearly designed for research data, where the term *data* was intended to denote *inputs or outputs of a processing*, that may or may not be automated depending on the scientific domains. As a consequence, FAIR principles have a strong focus on metadata. In principle, anything can be seen as data for a particular kind of processing: articles are data for Text and Data Mining, DNA strands are data for sequencing, software source code are data for vulnerability scanning tools, personal entertainment habits are data for recommendation algorithms. But if we look closer, it is easy to see that many of these objects are not *just data*, and most of them are *mainly not data*, in the sense that their intended use is not to serve as input for a processing pipeline. Looking at these objects as *just data* is therefore unsatisfactory, as it misses the key issues at stake. Software and source code are a clear example of this phenomenon, leading to several efforts to get the focus back on software as *not just data*. Following several years of work and discussion Lamprecht *et al*.^13^, Gruenpeter *et al*.^14^, Barker *et al*.^15^ introduced FAIR principles for research software (FAIR4RS). More recently, the Research Data Alliance (RDA) published a similar set of principles^16^ advocating for the improvement of sharing and reuse of research software, recently extended by Sonabend *et al*.^17^. All these efforts are laudable and will definitely help to make software a first class citizen in research. However, we believe that additional efforts can build upon the experience of FLOSS, taking into account the core problems of software complexity, but also the specificities of organization and purpose in scientific research. For example, the idea that “software reads, writes and exchanges data in a way that meets domain-relevant community standards” Barker *et al*.^15^ cannot be enforced when your work depends on closed-source software whose data formats cannot be easily changed. Actually, in some dramatic cases, this works the other way around: the “domain-relevant community standard” for human gene nomenclature underwent renaming of some core concepts to avoid issues with spreadsheet applications^18^. Similarly, the website fair-software.eu recommends registering code on a software registry in order to make it findable. Findability is an important problem, but we believe that equally important issues need to be addressed as well, namely being able to execute research software and collaborate on research software development.

### A tentative roadmap

To this end, we propose a comprehensive roadmap that takes into account all stakeholders in the research software ecosystem. While authors and contributors of research software remain pivotal, it is imperative to recognize the significant roles played by funders, research institutions, publishers, libraries, and policymakers: they have the means to make these new practices easier to apply and normative. Building on the guidelines of the Journal of Open Source Software, this roadmap is progressive, enabling its gradual adoption. This tiered approach is adopted for several reasons:

1. An overly stringent prescription can deter from engagement. The first tiers have the role of moving forward while not discouraging participants who might feel overwhelmed by seemingly unreachable goals.
2. The approach acknowledges the diverse backgrounds of scientist-developers, many of whom may not have formal training in software engineering but are deeply committed to scientific software development^19^.
3. It is difficult to impose stringent rules without appropriate recognition of the effort that they require: in the current landscape, with software development still underrated, this can be counterproductive.
4. The level of maturity with software practices in research vary significantly across disciplines and from country to country: a gradual roadmap seems the best way to bring everybody up to speed over time.

**Fig. 1:**
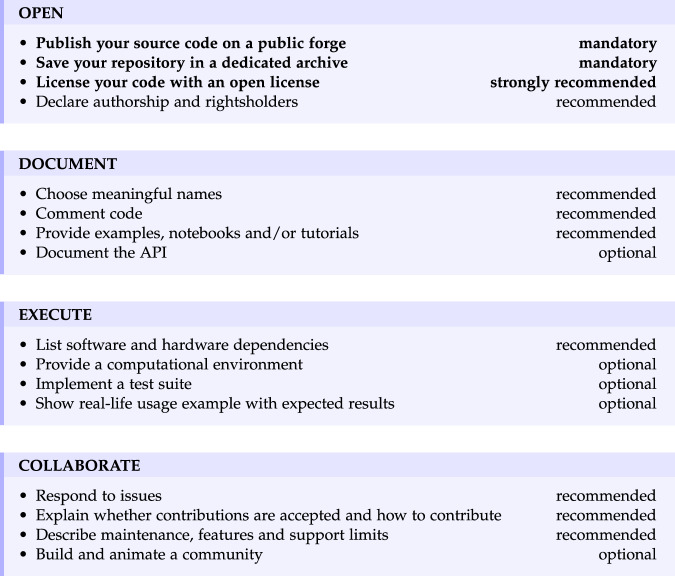
Summary of the CODE gradual road map organized in four categories: Open, Document, Execute and Collaborate.

**Fig. 2:**
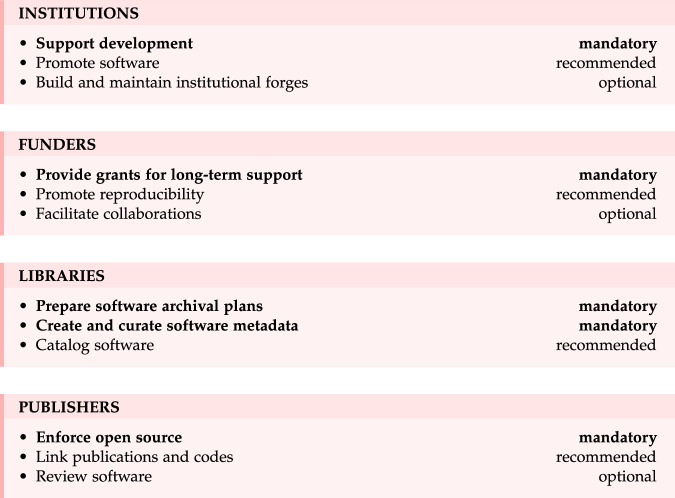
Summary of guidelines for the different stakeholders.

We detail the proposed roadmap that can be summarized as Open, Document, Execute, Collaborate, each element representing a key pillar for maximizing the value of research software in the realm of open science (see Fig. 1). Even though we do not necessarily aim to promote a specific acronym that may obscure the actual philosophy, it is to be noticed that CODE summarizes the key points of the roadmap quite well. This roadmap is then extended to propose a call for action from the main stakeholders, i.e., institutions, funders, libraries and publishers (see Fig. 2). A summarized version of this roadmap is also available as a commentary^20^.

## Results

### The CODE gradual roadmap for scholars who develop software

Figure 1 summarizes the CODE gradual roadmap, which is split into four categories, each containing four recommendations. We now expand on those recommendations.

### Open



#### Publish

There are many options for making software accessible to the community, but only a few of them are recommended and an even smaller number will pass the test of time. Distributing software upon request is not a good practice. It is very fragile since researchers can move from one lab to another, invalidating both their email and website. Additionally Collberg *et al*^21^, Stodden *et al*.^22^ found that email requests for software were not honored in the majority of cases. A safer option are forges: dedicated websites whose goal is precisely to facilitate the dissemination and collaboration around source code development, using version control tools such as git. At the time of writing, popular forge systems include Forgejo, Gitlab and the GitHub website. Version control represents a barrier for researchers not yet familiar with these tools, but this barrier is worth overcoming because forges provide some guarantee of durability and offer additional services, such as the preservation of the software’s development history, support for collaboration among developers, and interaction between developers and users.

#### Archive

Forges have become essential collaborative development platforms, but they are not archives. On the one hand, the owner of a project hosted on a forge may *alter, move or remove it at any time*. Document archives like Zenodo or Figshare are being used by researchers to store copies of specific versions of their own source code, but these archives are not software oriented: the version control history is lost, there is no control of granularity, and no intrinsic identifiers. On the other hand, and most importantly, *forges make no commitment to long-term maintenance*, independently of the financial clout of their owners: as a reminder of this fact, two popular forges were closed in 2015 (Gitorious) and 2016 (Google code), making all projects unreachable (over 1.4 million according to Software Heritage archive). The shutdown of such popular and well funded forges was a shock to many, and made clear the need for a *long term archive specifically designed for software* that addresses all the above issues, leading to the creation of Software Heritage, a non profit international multi-stakeholder initiative started in 2016 in partnership with UNESCO, which is today the *state of the art solution* to address the long-term availability of *all software source code* together with *its full development history*^23,24^. Software Heritage proactively harvests code hosting and distribution platforms, and provides mechanisms to trigger archival on demand. As of 2024, its archive contains over 22 billion unique source files collected from over 340 million projects, with all their version history, including full copies of the projects that were removed from Gitorious and Google Code. These copies can be used to repair the broken links in the web of academic knowledge. Software Heritage also provides a Software Hash persistent intrinsic identifier (SWHID) for all archived software artifacts. A SWHID allows to precisely pinpoint a version of source code at all levels of granularity^25^. Software Heritage provides the core infrastructural layer that links the academic ecosystem^26^ with the many other ecosystems that rely on software, such as industry and public administration^27,28^.

#### License

While most research software is shared publicly with the intention to make it accessible and reusable for all, following the principles of Open Science, very often this intention is not formalised by the explicit addition of a licence to the code or the repository. If the rightsholder wants to make possible the use of their software, a license that explicit the terms and conditions to this use is mandatory. Indeed, in the absence of a license, the rightsholder keeps exclusive rights (see Choose a license): even if the rightsholder has no intention to exercise them exclusively, everyone else will be legally forbidden from (re)using it, reducing its usefulness. Thus, we strongly recommend to provide a license, and preferably an open-source one for the sake of Open Science. The choice of licence should be made in consultation with affiliated institutions and project funders, ensuring at the same time compliance with institutional policies and with the principles of open science.

#### Authorship

The identification of authors and copyright holders is an essential part of opening software, both for its legal aspects and for citing software in journal articles. Authors and copyright holders may be individuals, groups of individuals, a collective entity (development team), an institution, etc. Ultimately, authors and copyright holders are the only ones who can define the conditions of reuse for a software, via a license, so it is important that they can be identified.

### Document



The audience for software documentation is diverse. It includes users, who need to understand what the software does exactly and how it is used correctly. It also includes current and prospective developers who wish to modify the software for different applications, and who need to be aware in particular of any assumptions built into the software that may not be valid for the application they have in mind. For larger and long-lived software packages, the audience for documentation includes current and future contributors and maintainers. And in preparation for a hopefully near future in which scientific software will be peer reviewed, the audience for documentation includes reviewers, who need to be convinced that the software is appropriate for the use case they are reviewing.

#### Choose meaningful names

Documenting code starts with naming variables and functions. When done wisely, these names convey a lot of information to the reader (including the author herself when she looks at her own code some months or years later). For example, naming a function da vs discrete-analysis makes a real difference in terms of understanding and re-usability. For short scripts, good names can be sufficient to make the code understandable.

#### Comment code

When names are no longer sufficient, comments added to the code become important. Their goal is not to rephrase the code, but to explain the reasoning that lead to the code. For example, if the code computes the square root of a number, that number must be positive, and a comment may be required to explain why the number is always positive in this specific context. Moreover, non-trivial algorithms require comments that provide a higher-level view of what is going on, to help the reader who might otherwise be lost in the technical details. The description of the parameters that have to be provided to a function, with their type and the reason for this type, is another example of a necessary comment.

#### Provide examples

Application Programming Interface (API) documentation is necessary for precision, but not always sufficient to help new users get started, in particular for large APIs. Learning how to use a complex software package from its API documentation is like learning a foreign language from a dictionary and a grammar. Beginners need pedagogical documentation, such as tutorials and documented usage examples.

#### Document the API

For larger software packages, users cannot be expected to read the code in order to understand what it does and how to use it. Code readability and good comments still matter, for the benefit of contributors, but users require a separate document that focuses on the API that they interact with. API documentation lists and describes the functions, classes, shell commands, and other entities in the software that users refer to in their own code that orchestrates the computation at a higher level.

The transition from commented code to a software package with an API documentation is a major one. Users of commented code have to read and understand the code, and adapt it to their own needs by modifying it. Users of a software package with an API expect to learn everything they need to know from the documentation, and don’t even attempt to read the code. Much scientific software is in a dangerous intermediate state: there is an API documentation, but it may not be up to date, differ from the actual behavior of the code, or be incomplete, leaving out details that can be crucial for some applications.

### Execute



#### List software and hardware dependencies to avoid dependency hell

Running software requires a computational environment, which consists of a hardware (the computer) and other software items, called the software’s dependencies. If you wish to run a piece of software published by someone else, you must figure out how to construct a suitable environment, by starting with a compatible computer system and installing all the software’s dependencies. Unless an explicit list of dependencies is supplied, this task may turn out to be prohibitively difficult or time consuming. This is why we strongly recommend authors to provide a complete and precise description of the dependencies with each published piece of software. Unfortunately, providing such a description is not always easy. You may be able to figure out the components of the environment that you use yourself, although even that can be a difficult task. But not all the components of your environment are actually required. If you run a piece of software on a desktop computer, there is a good chance that it has a Web browser installed, but it is unlikely that this Web browser is required to run the software under question. It is also likely that the software will work equally well in an environment that has somewhat different versions of the dependencies. These uncertainties make it difficult for software authors to describe the environmental requirements, and even more difficult for software users to set up an environment that conforms to these requirements. In the worst case, users succeed in constructing an environment in which the software works without obvious problems, but produces different results than in its original environment. For example, Bhandari Neupane *et al*.^8^ found a portability bug in a previous work, where a research software provides incorrect results in some operating systems. We also refer the reader to a recent initiative to list errors due to research software^29^. Dependency issues and uncertainties are one of the root causes of computational irreproducibility^30^.

#### Provide a computational environment

One way to help users set up a suitable environment is to provide either an archived environment in the form of a container image (using Docker or virtual machines), or a recipe for constructing an identical environment for a reproducible software manager, such as Guix or Nix. Both approaches require technical expertise that many researchers do not have, which is why we consider this step optional within the current state of the art. A good overview to get started writing Guix recipes is given in the document A guide to reproducible research papers. Such recipes are the technically best way to provide the description of a computational environment: they are small text files that are easy to publish and archive, and they can be easily modified to explore alternative computational environments for the software. In contrast, container images, created and run using container management software such as Docker or Apptainer, are large files that can be used as-is, but not easily modified. rworkflows Schilder^31^ is an example for providing both an easy and an advanced solution, for statistical and social sciences and related fields.

#### Implement a test suite

Given the inevitable variability of computational environments, another helpful step is to provide a test suite for the software. A test suite consists of simple example use cases for the software for which the correct results are known and recorded. Executing the test suite verifies that the results are indeed the expected ones, providing the user with reassurance, though not proof, that the software works correctly in its current environment. These test suites are also highly helpful for the original software developers, in order to detect breaking changes early in their development process.

#### Real use cases

A final optional step for helping users to get started with running the software is a collection of usage examples from real-life applications, with the expected results. We have already mentioned examples as documentation, but examples become much more valuable if they are executable. Such examples differ from tests in two ways: first, they are meant to be studied and modified by the user, whereas a test suite is typically executed blindly. Second, they illustrate common usage scenarios of the software, whereas tests tend to focus on simple scenarios and on edge cases that are important for correctness even though they rarely occur in practice.

### Collaborate



Traditionally, collaboration in research means that a group of researchers works together on a research project and in the end publishes a joint paper, of which everyone who contributed is a co-author. Such collaborative research can include the production of software, usually in the form of scripts and notebooks that are specific to a project. That is not the kind of collaboration we are discussing in this section. We focus on *open* collaboration that anyone interested can participate in. Open collaborations are typically long-lived and broad-spectrum efforts, which in the realm of software means tools of interest to entire communities. However, not all research code is expected to build and sustain collaborative environments. In practice, collaboration starts by being explicit about what can or cannot be expected in terms of support and maintenance, and whether contributions to the code are welcome.

#### Respond to issues and/or feature requests

The first level of open collaboration on a software package is inviting feedback. Are there any problems with the software? Does it behave incorrectly, or is it too difficult to use? Is there functionality that could be usefully added to it? Issue trackers in software forges were designed to collect such feedback and support open discussions about them.

#### Explain whether contributions are accepted and how to contribute

The second level of open collaboration is permitting and soliciting not only feedback, but also contributions, be it to the code itself or to its documentation, or to the governance structure of the project. This requires careful thought. Contributions need to be reviewed, which takes time. Contributions that make the code larger or more complex increase the maintenance effort in the future. It is therefore necessary to decide on a policy for handling contributions, define a workflow, and document it publicly. Furthermore, it is good practice to think about *onboarding*, i.e. integrating new contributors into the collaboration. For example, many projects maintain a list of easy problems (“good first issue”) that are suitable as entry points to become familiar with the code and with the culture of its community.

#### Describe maintenance, features and support limits

The experience with FLOSS projects has shown that when a piece of software attracts a critical mass of users, such feedback can easily overwhelm the development team. After all, criticism (even when fully justified) is easy, whereas dealing with it is a lot of work. An important step for open collaboration is thus defining limits and stating them clearly. What level of maintenance and support are the developers able and willing to provide? Which kind of feature requests will be considered, and which will be considered out of scope?

#### Build and animate a community

The third level of open collaboration is to build a community around the software. This involves organizing tutorials, workshops, and hackathons, and mentoring new contributors. For very large projects, it can also involve communicating to a wider public, and establishing relations with decision makers in industry or society. The goal of community building is not growth for growth’s sake. Success of a community should rather be evaluated by the satisfaction of its members, its willingness to welcome everyone who wishes to participate, and its integration into a wider scientific ecosystem that ensures epistemic diversity and critical evaluation of each other’s work.

### A call to action for all research software stakeholders

Besides researchers and engineers, there are several stakeholders that have prominent roles to play in supporting scientific software in all its dimensions. Researchers and engineers are the main actors, but they require support and recognition for their work, both of which are still lacking in a large number of countries and institutions. Knowles *et al*. ^32^ further discuss this issue. Their dramatic example concerns the ehtim software, which contributed to making headlines through the first image rendering of a black hole in 2019, while the authors were at the same time denied funding to maintain it. The fragility of software dependencies and maintenance is also masterfully captured by XKCD #2347.

### Institutions



#### Support Development by Investing in the Required Human Resources

Nowadays, software development is a prevalent part of scientific work. As such, we advocate for first-class recognition and support of software development activities by institutions. This support is provided by various means. The primary mean is to hire research software engineers, who bring the necessary expertise to develop, maintain and deploy research software. This need is not new: in many countries research engineers working on software have been around for decades, well before the recent grassroot effort that led to the creation of research software engineer societies, but it is important to recognise them as a specific body, with skills and requirements of their own. These research software engineers are the backbone of the software development process and can help raise awareness of basic software development practices among research staff^33^.

#### Support Development by Creating Legal Frameworks that Scientists and Engineers can Find their way around

A team wishing to publish a piece of software must be able to find out easily who the rightsholders are and which person to contact for inevitable legal arrangements such as choosing a licence. Ideally, institutions should make it possible for scientists and engineers to publish research software under an Open Source license without imposing a lengthy bureaucratic procedure for obtaining permission.

#### Promote Software

Institutions can help promote software by acknowledging software creation and development as contributions that can be taken into account when hiring or promoting scientific and engineering staff^34,35^. The creation of software-related awards can create visibility and awareness on the importance of software development within institutions. To the best of our knowledge, France has led the way by creating a national Open Science Research Software Award^36^ in 2022. These prizes reward projects and teams working to develop and disseminate open source research software, and are presented by the Ministry of Higher Education and Research in a high-profile annual ceremony. The award has several categories aimed at showcasing projects according to their scientific and technical dimensions, their capacity to train and lead a community and the quality of their documentation. A full description of the goals, difficulties, approach and lessons learned has been published by Catala *et al*.^37^, providing actionable blueprints for a broader adoption of the concept. New research software awards have been recently created, for example with the South African National Science and Technology Forum^38^.

#### Build and maintain institutional forges

Software forges were created some 25 years ago as a means to facilitate collaboration within developer teams. With the fast growth of Open Source, and the advent of modern version control tools, some of them have progressively become large scale social networks, with the associate network effects that lead to concentration into a handful of big players. Research software developers face a difficult decision about which forge to choose, based on the target audience and network. The options range from institutional forges (i.e. hosted on institutional servers) to publicly available, general purposes commercial ones (i.e. GitHub, GitLab, BitBucket), with open-source community forges as an alternative if the software fits the requirements of such a community. Le Berre *et al*.^39^ present a comprehensive description of the issues at stake when building institutional forges, based on a broad analysis of over 70 such forges used in French Higher Education and Research. One key issue is the tension between the need to maintain control over the use of the resources, and the desire to enable contributions with minimal friction from unknown individuals worldwide. Currently, access to most of the analyzed forges is available only to the institution’s own members, which drastically limits external contributions, and even becomes an obstacle for the automated workflow of journals dedicated to software, such as JOSS, which requires submissions to be available on a forge which is open to anybody (without registration approval). It seems that this is the reason why so much software is currently developed on commercial forges. This situation may change if a coordinated effort is put in place to remove the technical and administrative obstacles that make it currently difficult to accept external contributions on institutional forges (e.g. via federation of identities and appropriate access control to the different resources).

### Funders



#### Provide grants for long-term support of valuable software

As recently reiterated by Coelho^40^, *most [research] software is designed and built by research groups funded by short-term research grants*. These short-term grants are both beneficial and detrimental to a software project. They are beneficial for seeding new software through temporary hirings, and sometimes start building a community. They are detrimental because once the grant is finished, researchers are on their own to maintain the software to the best of their skills. Maintaining software is difficult and requires advanced skills in software development to deal with software collapse^41^. Thus, we believe that public research funders should additionally offer grants to support long-term maintenance of software with a proven value (e.g, used and cited by the scientific community), and not only development of new tools and systems. This issue has been acknowledged by the Research Software Alliance Funders Forum recently. Pioneering public research agencies have recently proposed the first software maintenance grants, see for example the UK’s Software Sustainability Institute or the German Research Foundation. The private Chan Zuckerberg Initiative has shown one way to do this with its *Essential Open Source Software for Science* program: we believe more research should go into exploring the different mechanisms that can be put into play to balance between funding innovation and supporting maintenance with the necessarily limited resources that are available^42^.

#### Promote reproducibility

Being able to reproduce and replicate results is a cornerstone of the scientific approach. However, the scientific community is facing a reproducibility crisis^43–46^. Funders should ensure that by default, research software is licensed and distributed under free and open-source licences. Limited exceptions can then be introduced when closed source code is necessary, e.g. for security concerns, confidentiality, or in some industrial collaborations. This continues a tradition where important funders, such as the European Research Council, have successfully been promoting key open science practices (e.g., open access publication and open data).

#### Facilitate collaborations

We have explained previously that scientific software depends on a software stack whose components have very diverse origins. These components can be themselves highly specialized scientific software, but they can be also much more generic and serve broader goals. To name but one example, the ubiquitous NumPy library Harris *et al*.^47^ is *the primary array programming library for the Python language* and *has an essential role in research analysis pipelines in fields as diverse as physics, chemistry, astronomy, geoscience, biology, psychology, materials science, engineering, finance and economics*. This library is community driven and its development *still depends heavily on contributions made by graduate students and researchers in their free time*. This situation is problematic because it means a large part of science depends on the free time of a few people. This is the reason why funders need to consider alternative funding models, where, for example, a subset of a grant could be dedicated to the support of a core library or a core developer could be supported through the grant even though her work would be only indirectly linked to the grant. Research funders could copy recent initiatives applied to FLOSS, such as the publicly-funded German Sovereign Tech Fund.

### Libraries



#### Prepare software archival plans

As we have explained before (“Archive” recommendation for scholars who develop software), while forges are great tools fostering collaborations, they are not suitable for archiving software. Libraries are dedicated to archiving scientific work, and have recently engaged in promoting open science practices^48^. We suggest that libraries should go one step further and create software archival plans, ensuring that local scientists properly archive their research software hosted on forges (be it local or global ones).

#### Create and curate software metadata

Libraries have historically created and handled metadata and citation systems for books and articles; we argue they should develop similar processes for software. Software is a very complex and fragile object and therefore, metadata is needed for providing provenance information as well as to properly credit authors. One first kind of metadata is persistent identifiers. For example, Software Hash Identifiers (SWHID) are permanent identifiers that allow to reference a specific version of the source code of a project, at different levels of granularity. A second kind of metadata can be required to cite software, for example using CodeMeta, Bioschemas ComputationalTool or the Automated Software Metadata Publication. Indeed, citing the article mentioning a software package is common practice among researchers but is not compatible with the software lifecycle, which makes this citation eventually inaccurate. This is the reason why Chue Hong *et al*.^49^ highlighted the need to cite the software itself, acknowledging is as a legitimate product of research. It is to be noted that referencing and citing are related to distinct needs. Citing software refers to the authorship: the purpose is to attribute credits.

#### Catalog software

Once software is well-described through appropriate, potentially curated metadata, we can implement the Findable principle of FAIR by creating software catalogs. These catalogs can be handled by libraries at an institutional level to showcase contributions and productions (NASA’s Software Catalog, French Catalog for Research Software), or for specific scientific communities (swMATH, bio.tools).

### Publishers



#### Enforce open source

Following the wide adoption of open science practices scientists and publishers, we propose to tackle the reproducibility crisis by enforcing the open-source publication of code alongside papers. *Ugly* code is always better than no code^50^. Sharing code has been made extremely easy with Zenodo or Software Heritage, even though it still requires a minimal experience with version control tools. In that context, publishers have a role to play in order to guarantee that code is not only promised, but actually shared at the time of publication. We cannot be satisfied anymore with the current recommendations according to which *availability upon request* is still an option. Case in point, the Nature journal received severe criticism^51^ from the machine learning community, following the publication of McKinney *et al*.^52^. Haibe-Kains *et al*.^51^ explains that the lack of details of the methods and algorithm code undermines its scientific value. This criticism lead to a policy change for the given journal.

#### Link publications and codes (and data)

In order to make research more transparent, accessible and inclusive, it is essential to have access not only to publications but also to all scientific inputs used to create them them, and in particular code and data. Publishers have a significant responsibility for ensuring that all these scientific objects are linked together. The publication ecosystem must now integrate sources code. Some editors have already started this process. For example, Dagstuhl Artifact Series (DARTS)^53^ and IACR’s Artifact Archive^54^ enable publication of peer-reviewed software artifacts (cf. next paragraph) alongside papers that have been accepted in a conference or journal from the same editor. Long-term archiving of source code can be obtained by interoperating with code archives such as Software Heritage.

#### Review Software

As described in detail by Di Cosmo *et al*.^28^, Informatics Europe *et al*.^55^, various communities have over time put in place mechanisms for reviewing software associated to publications, ranging from a simple check of existence, to quality of metadata, to quality of software and adequacy with respect to what is stated in the publications. As an example, some computer science communities – including the formal methods, software engineering, systems, security, and architecture communities – have put in place an *artifact evaluation* process, with a dedicated program committee, which started in 2011. In these communities, authors are encouraged to submit a software artifact in addition to their paper. Artifacts are built and evaluated to ensure that all the software-related experimental claims of the paper can be reproduced by anyone. An artifact consists of a snapshot of the computational environment and the software used for the experimental evaluation of the paper – distributed for example as a docker container or an image of a virtual machine. Reviewers may grant different badges to an artifact, depending on its various qualities^56^. Publishers should consider getting together to identify the various levels of review that have been field-tested, and propose a unified approach to enable the various research communities to adopt the one most appropriate to them.

## Discussion

Not all scientific software is created equal. Some is meant to be widely disseminated in the scientific community, to grow and to be maintained in the long term, while other software serves solely as a one-shot illustration of a concept or an idea. But both are important for science. Independently of the goal, the size, or the form of the software, we advocate first and foremost to share the code and archive it permanently, even if authors may think it is not worth being shared. Then, and only then, more can be asked from the authors. Practices currently vary widely across the scientific community. Much research software is still not published at all, but some well-established open-source research software projects already follow all our recommended steps.

In their FAIR4RS principles Barker *et al*.^15^, extend the FAIR principles to the case of software, in order to improve how to find it, how to access it, how to interoperate software, and how to reuse software. Only two years after its publication, it has been adopted internationally by various communities. FAIR4RS includes recommendations “R1.1. Software is given a clear and accessible license.” and “R3. Software meets domain-relevant community standards.”, which are related to some of our CODE principles for software developpers. The goal of CODE is to provide additional guidance to improve research software distribution and reuse. In addition, scientists cannot bear these new aspects of scientific research alone: CODE also includes a call to action for other research software stakeholders to appreciate and fund software. Contrary to some communities who adopted FAIR4RS, we believe in particular that the long-term sustainability of software should *not* be the sole responsibility of scientists.

^Alves, R. *et al*.57^ introduce Software Management Plans, providing a methodology for expert computer scientists to gradually improve their software. Alves *et al*.^57^ mention that “there are a few SMPs already available, most of them require significant technical knowledge to be effectively used”, although recent advances aim at making them more accessible^58,59^. Our aim in this work is to provide a tiered roadmap to ensure minimal good practices will be followed by non-expert scientists. In addition, our work advocates for actions put in place by all stakeholders of the scientific process.

Institutional support and explicit open-source policies are gaining traction. Some of these recommendations have already been put into practice. To name a few, several countries now regularly award outstanding research software^60^. Leading institutions, including NASA through its Open Science Program and Open Source Funding Program (2024) and the international Coalition for Advancing Research Assessment, recognize the value of software engineering in research. Research institutes like France’s National Institute for Research in Digital Science and Technology (INRIA) consider software development, in addition to as other markers of achievements such as publications and community service, when hiring^34^. At both the European and international levels, a number of actors are also present and work similarly for the recognition of scientific software in science. To name just a few, we can cite the Research Data Alliance, UNESCO, Software Heritage, the Research Software Alliance, the European Open Science Cloud, Knowledge Exchange and Zenodo. Members of the EVERSE project have recently surveyed the “rewards and mechanisms for research software and training activities”^35^. Private foundations, such as the Sloan Foundation and the Chan Zuckerberg Initiative in the USA, have been engaged with the recognition, support and promotion of scientific software for a few years already, with policies that start to have long lasting impacts along several dimensions.

We are happy to see these improvements, but there is still a long way to go. First, we think that what is holding researchers and engineers back is mainly a lack of support from institutions, funders, libraries and publishers. Good open-source software requires long term resources: good infrastructure, secured funding and training. Second, it is time to recognize research software as a valuable scientific contribution in itself, beyond a tool – and to support it. It is important to re-assess that research software is special: it has become over the years a critical ingredient of science and yet, it is still not recognized widely enough as a valuable scientific contribution^61^. It cannot be reduced to “just data”, and imposing some high-level principles that ignore this fact might be counter-productive: FLOSS developers and researchers have created and shared code for many decades and their experience and practice, formed well before the term Open Science became popular, must be properly taken into account.

The CODE gradual roadmap for scholars who develop software shares some goals with the process of “artifact evaluation” started by some computer science communities in 2011^62^ – although artifacts and research software are not equivalent. After a few years, a group of academics related to the Association for Computer Machinery (ACM) standardized artifact evaluation criteria through a badging system^56^. Note that other associations such as EAPLS use similar systems, and that non-ACM publisher such as Dagstuhl’s DARTS/LIPIcs use it. The “Artifact Available” badge mandates that the artifact is publicly accessible and archived (usually on Zenodo). The “Artifact Evaluated: Functional badge” mandates that the artifact is documented and exercisable with included scripts to generate the results in the paper, which is similar to some of the recommended points of the Document and Execute blocks. The “Artifact Evaluated: Reusable” badge mandates that the quality of the artifact significantly exceeds minimal functionality, which is close to some points of the Document and Execute blocks.

## Methods

We gathered as a diverse group of experts in Scientific Data and Research Software, sharing an interest in Open Science and Reproducible Research. We started this work by studying the specificity of research software (compared to research data), and the consequences of this specificity on research software. We made sure our discussion group was interdisciplinary in order to propose principles that would apply broadly and be understandable to every scientist, not only specialized computer scientists. This work is the culmination of three years of discussion within this group.

### Conclusion

Research software is now a foundation of most scientific research. This article presents the CODE gradual roadmap for scholars who want to improve their research software, thus contributing to improving scientific reproducibility. This roadmap is complemented by a call to action for research software stakeholders to truly make research software a pillar of the scientific process.

## Data Availability

No data is shared in this paper.

## Code availability

No code is shared in this paper.
